# Tubulointerstitial nephritis and uveitis (TINU) syndrome: a systematic review of its epidemiology, demographics and risk factors

**DOI:** 10.1186/s13023-017-0677-2

**Published:** 2017-07-14

**Authors:** Linda O. Okafor, Peter Hewins, Philip I. Murray, Alastair K. Denniston

**Affiliations:** 1 Department of Ophthalmology, Queen Elizabeth Hospital Birmingham, University Hospitals Birmingham NHS Foundation Trust, Birmingham, UK; 2 Birmingham & Midland Eye Centre, Sandwell & West Birmingham Hospitals NHS Trust, Birmingham, UK; 3 Department of Renal Medicine, Queen Elizabeth Hospital Birmingham, University Hospitals Birmingham NHS Foundation Trust, Birmingham, UK; 4 Institute of Translational Medicine, Centre for Rare Diseases, Birmingham Health Partners, Birmingham, UK; 5 Institute of Inflammation and Ageing, Academic Unit of Ophthalmology, University of Birmingham, Birmingham, B15 2WB UK; 6 NIHR Biomedical Research Centre at Moorfields Eye Hospital and UCL Institute of Ophthalmology, London, UK

**Keywords:** Tubulointerstitial nephritis and uveitis syndrome, TINU, Tubulointerstitial nephritis, Uveitis, Inflammation

## Abstract

Tubulointerstitial nephritis and uveitis (TINU) syndrome is a rare oculorenal inflammatory condition that was first described in 1975. In 2001 a major review identified 133 cases in the world literature and proposed key diagnostic criteria for the condition. Although acknowledged as rare, the limited data available prevented reliable estimates of the prevalence of the condition, and hampered elucidation of the relationship between genetic and environmental factors that contribute to its pathogenesis.

In this review we have performed a systematic search on the epidemiology, demographics and proposed risk factors for TINU. Estimates of prevalence based on studies that explicitly report TINU cases suggest that it is diagnosed in 0.2–2% of patients attending specialist uveitis services, with variation reflecting a number of factors including level of diagnostic certainty required. The prevalence of uveitis in patients with tubulointerstitial nephritis (TIN) may be higher than currently recognised, particularly in the paediatric population.

The prevalence of TINU is higher in younger age groups and there is a female preponderance although this gender effect appears weaker than suggested by early studies. Although important genetic contributions have been proposed, the small size of studies and variation between reports currently preclude identification of a ‘pro-TINU’ haplotype. Drugs and infections have been proposed as the leading acquired risk factors for the development of TINU; whilst the small size of TINU cohorts and issues of study design limit interpretation of many studies. Larger datasets from the renal literature suggest that the majority of these cases are precipitated by a drug-induced hypersensitivity reaction; however in many ophthalmic cases no clear precipitant is identified.

## Background

Tubulointerstitial nephritis and uveitis (TINU) syndrome was first described in 1975 by Dobrin et al. [1]. A comprehensive review published in 2001 identified 133 cases in the world literature and proposed diagnostic criteria for this entity [2]. It is defined as the occurrence of tubulointerstitial nephritis (TIN) and uveitis in a patient in the absence of other systemic diseases that can cause either interstitial nephritis or uveitis; it is therefore a diagnosis of exclusion [2, 3]. TINU is thought to be an immune mediated process that may be precipitated by drugs or infections, although in many cases no cause is identified (idiopathic) [2]. Most series suggest that TINU only accounts for 0.1–2% of patients seen in specialized uveitis centres but the syndrome is likely to be under-diagnosed [2, 4]. Given that over half of all uveitis cases have no identified cause it is pertinent to consider TINU in undifferentiated cases of uveitis, and to be aware of its possible associations with common systemic medications and infections. The challenge of diagnosis is in part compounded by the heterogeneity that exists within the uveitis spectrum. Although all forms of uveitis are characterised by intraocular inflammation, the symptoms and signs seen vary according to the primary site of inflammation within the eye (the anatomical subtype of uveitis). Most patients with TINU experience a bilateral sudden-onset anterior uveitis which presents with typical symptoms of redness, pain and photophobia. It is becoming clear, however, that this is not the only uveitic phenotype associated with TINU and that ophthalmologists need to remain alert to the possibility of TINU in the context of other clinical presentations of uveitis.

TIN itself is a potentially life-threatening condition, accounting for up to 15% of cases of acute kidney injury (AKI) and is characterised histologically by interstitial oedema with inflammatory cell infiltrates and tubular damage [5]. TIN should be considered in the differential diagnosis of all patients with unexplained AKI or progressive reduction in glomerular filtration rate (GFR). The urine sediment may be bland or active (denoted by the presence of red cells and red cell casts). Tubular proteinuria may be detectable but high levels of albuminuria are usually absent because glomerular pathology is not prominent. A proportion of patients with acute interstitial nephritis (AIN) exhibit sterile pyuria. Patients may present with non-specific constitutional symptoms including fever, rash, joint pain, malaise or flank tenderness or be asymptomatic and detected through abnormal renal function (estimated GFR) tests. A proportion of patients develop peripheral blood eosinophilia but this is in an inconsistent feature. Similarly, urinary eosinophilia may be detectable in some patients but this abnormality is not evaluated in routine laboratory tests at most centres. A renal biopsy is required to confirm the diagnosis. It is also important to exclude systemic diseases known to cause a similar overlap of ocular and renal inflammation, notably sarcoidosis, Sjogren’s syndrome, systemic lupus erythematosus (SLE), and tuberculosis (TB).

In this review we consider the available evidence regarding the epidemiology of this rare condition, and critically appraise the evidence underlying the current understanding of both genetic and environmental risk factors. Furthermore, it is recognised that the challenges faced in assessing incidence and prevalence in TINU are replicated across many other rare diseases, including many sight-threatening forms of uveitis (such as birdshot chorioretinopathy and punctate inner choroidopathy). A critical and systematic approach to the evidence around all such rare uveitis syndromes is needed, both to clarify what is known but also to highlight areas where there is currently a major evidence gap.

## Methods (search strategy)

The original literature search was undertaken in January 2016, with an updated search conducted in May 2016 to identify any ‘late-breaking’ articles. The following databases were searched: Medline, EMBASE, and the Cochrane Library with a date limit of 1946 to the present for Medline, 1974 to present for EMBASE and no date restriction for Cochrane. The search used the following terms: “Tubulointersititial nephritis and uveitis”, “TINU syndrome” and related terms resulting in 498 identified articles; 252 articles were excluded on the basis of being duplicates (225) or not directly relevant (27). In order to provide greater information on the lower end estimates of prevalence and incidence of TINU it was important to include studies that described large cohorts of uveitis patients in whom no cases of TINU were reported. To achieve this additional searches were undertaken using the terms: “Uveitis” and “prevalence OR incidence” with identification of studies containing more than 500 patients (250 for paediatric cohorts), and supported by hand-searching of the bibliographies of relevant studies.

All relevant clinical studies were considered but were weighted according to their level of evidence, in which well-designed randomized prospective clinical trials would be ranked highest and case-reports ranked lowest (excluding expert opinion); case-reports were generally excluded from the final review unless they were considered to provide unique insights not evident from higher level studies. Articles which did not present primary data (such as reviews and expert opinion) were also considered and were included if they provided original insights into the condition, based on appropriate published primary data. In addition their bibliographies were hand-searched to identify any relevant additional articles. There were no language restrictions on this review.

## Epidemiology

### Global estimates of prevalence

TINU is a rare condition, and estimates of its prevalence within patients attending specialist uveitis services range from <0.1% to 2% in ‘all age’ populations and up to 2.3% in paediatric populations (Table 1). Nevertheless, the data is limited and does not allow more accurate estimates. Of the larger surveys of ‘all age’ uveitis services, the highest estimates come from Oregon (USA) where in 1988 Rosenbaum reported five patients with bilateral anterior uveitis and renal disease (three of whom had histologically confirmed interstitial nephritis) [3], and reported that TINU was diagnosed in 1.7% of uveitis patients attending his clinic [6]. Interestingly almost two decades later Mackensen et al. reported on TINU in the same uveitis service over the period 1985–2005 and again found a prevalence of 1.7%, representing 33 of 1985 patients [7]. The 2009 multicentre epidemiological survey in Japan noted a prevalence of 0.4% (*n* = 15) in 3830 patients with uveitis attending specialist uveitis services [8]. In Manchester (UK) Jones reported a prevalence of 0.2% (*n* = 7) in 3000 uveitis cases seen between 1991 and 2013 [9]. The UK cohort from Jones et al. is of particular interest as it also provides an estimate of overall incidence for referral to their uveitis service, allowing an estimate of the incidence of diagnosed TINU as around 1 per 10 million population per year for their region of the UK [9]. Most series that report any cases of TINU, report prevalence between 0.2–0.6% [8–12]. However there are a number of large surveys that report no cases of TINU (equating to a prevalence within their uveitis services of <0.1%).

**Table 1:** Studies since 1990 reporting on distribution of uveitis types

Author	Data range	Country	Design	TINU cases/ population(n)	TINU cases (%)
‘All-age’ Studies with >500 patients					
Rodriguez [47]	1982–1992	USA	Retrospective	0/1237	0
Rothova [48]	1984–1989	The Netherlands	Retrospective	0/865	0
Mackensen [7]*	1985–2005	USA	Retrospective	33/1985	1.66
Mercanti [49]	1986–1993	Italy	Retrospective	0/655	0
Oruc [50]	1990–1995	USA	Retrospective	0/853	0
Tran [51]	1990–1993	Switzerland	Prospective	0/558	0
Jones [9]	1991–2013	UK	Prospective	7/3000	0.23
Kotake [52]	1994	Japan	Retrospective	4/551	0.73
Barisani-Asenbauer [53]	1995–2009	Austria	Retrospective	0/2619	0
Rathinam [54]	1996–2001	India	Retrospective	0/8759	0
Singh [55]	1996–2001	India	Retrospective	0/1233	0
Yang [56]	1996–2003	China	Retrospective	0/1752	0
Soheilian [57]	1997–2000	Iran	Retrospective	0/544	0
Jakob [12]	2001–2006	Germany	Retrospective	10/1916	0.52
Al Dhibi [58]	2001–2010	Saudi Arabia	Retrospective	0/888	0
Kazokoglu [59]	2004	Turkey	Prospective	0/761	0
Ohguro [8]	2009–2010	Japan	Prospective	15/3830	0.39
Gonzalez Fernandez [60]	2012–2013	Brazil	Prospective	0/1053	0
Total				69/33059	0.21
Paediatric Studies with >250 patients					
Smith [61]	1980–2005	USA	Retrospective	12/527	2.28
Kump [4]	1985–2003	USA	Retrospective	3/269	1.12
BenEzra [62]	1989–1999	Israel	Retrospective	4/275	1.45
Paroli [63]	1995–2004	Italy	Retrospective	4/257	1.55
Total				23/1328	1.73

For inclusion a minimum number of 500 was required for ‘all age’ cohorts and a minimum number of 250 patients for paediatric cohorts. As discussed in the text, it should be recognised that studies which report 0 cases of TINU may do so due to (1) a true rarity in that population, (2) non-reporting of diagnosed cases (eg studies marked with a ‘+’ include the listing of ‘other diagnoses’ which could potentially include TINU cases), or (3) underdiagnosis

*Includes ‘possible’ as well as ‘probable’ and ‘definite’ cases

This variation between series may come from a number of factors which we classify as: *true differences between the population* (genetic and/or environmental): *true differences between the samples* arising from the nature of the uveitis service (paediatric, adult or both; secondary or tertiary; state or private); or *measurement differences between the sample*s (diagnostic criteria used; the degree of missed diagnosis; design of the study); or *reporting differences between the samples* (ie cases are diagnosed but are simply labelled as ‘other’ in reports of uveitis prevalence). Although there does appear to be a true difference in prevalence between certain populations which may be linked to genetic susceptibility (discussed later), much of the discordance between series may also arise from the influence of these other factors as outlined below. Recognition of these factors are important as they will affect any estimates of global prevalence: unrestricted aggregation of all series in Table 1 would suggest a prevalence of 0.2% in ‘all age’ uveitis services whereas this rises to 0.6% if only those studies that reported at least one TINU case are included.

#### Impact of missed diagnoses on estimates of prevalence of TINU

Although originally described in 1975 [1], TINU has received much greater attention from the late 1980s. It is likely therefore that more recent series e.g. from the 1990s onwards provide more accurate prevalence figures, although clearly expertise in this area was present in certain centres much earlier [3, 6]. Since patients with TIN may be asymptomatic or exhibit nonspecific symptoms (fever, abdominal pain) that do not lead to kidney function tests being performed, diagnosis of TINU may be significantly delayed or still unrecognized even after the onset of uveitis symptoms and ophthalmological assessment [13, 14]. Even when both the uveitis and renal disease are symptomatic, they may not be synchronous and so the connection between them may be missed. The review by Mandeville noted that ocular symptoms were concurrent with systemic symptoms in only 15% cases; in 21% cases uveitis occurred before systemic symptoms, occurring up to two months beforehand; in 65% cases, uveitis occurred after systemic symptoms with a median of 3 months, but noted up to 14 months [2].

It is often suggested that TINU is under-recognised and that most incidence and prevalence figures are likely to be under-estimates. The study from Mackensen et al. provides data to support this [7]. They identified that in the cohort of 1985 patients, 26 had been diagnosed with TINU during routine care (prevalence of 1.3%) however a further 7 patients who had been labelled idiopathic were consistent with TINU based on the criteria of typical bilateral sudden onset anterior uveitis with renal dysfunction (total prevalence of 1.7%) [7]. They also identified that there were a further 18 ‘idiopathic’ paediatric cases in which the uveitis was typical but in whom there had not been adequate laboratory investigations to rule in or rule out the diagnosis, leading to the possibility that the real prevalence is even higher [7].

Much of the previous discussion considers the identification of TINU cases from the ophthalmic perspective i.e. in a cohort of patients with established uveitis, how many have TINU? Equally important is to consider the renal perspective: in a cohort of patients with AIN, how many have associated uveitis? ‘Missed’ diagnoses may arise due to a failure of ‘diagnosis’ or a failure of ‘connection’. Just as studies show that even where concurrent renal disease has been diagnosed it may not have been appreciated by the ophthalmologist [7], so it is likely that there may be cases of AIN in which uveitis is either not diagnosed or is diagnosed but not connected (the patient does not deem the concurrence relevant so that the renal physician and ophthalmologist may not become aware of the other condition). One potential concern is whether uveitis may be missed because it is asymptomatic. In a retrospective review of 26 children with biopsy-proven TIN in Finland, uveitis was diagnosed in 12/26 (46%); uveitis was asymptomatic in 7/12 (58%) [15]. Interestingly, when a prospective study was conducted in the same population with regular slit-lamp examination (at onset of TIN, and at 3 and 6 months afterwards), a remarkable 16/19 (84%) were found to have uveitis; in 9/16 this was either diagnosed as part of the baseline examination or had already been diagnosed within the preceding month [16]. It is not clear whether these very high rates are specific to this young Finnish population, or whether there are indeed high rates of asymptomatic uveitis that go unnoticed and undiagnosed in the TIN population.

#### Impact of diagnostic certainty on estimates of prevalence of TINU

In addition to highlighting ‘missed cases’, the Mackensen study also illustrates how the level of diagnostic certainty required will impact the reported prevalence [7]. The study explicitly includes 13 possible and 7 probable cases, as well as 13 definite cases (classified according to the modified criteria of Mandeville et al. which are discussed later [2]), so providing a maximal prevalence in that cohort. It is worth noting that if only the definite cases were included then the prevalence within this service would have been 0.65%, more similar to other series.

## Demographic factors

### Younger age as a risk factor for TINU

The age group studied will also significantly affect the reported prevalence. TINU is predominantly seen in younger patients. In the review by Mandeville et al. of 133 patients gathered from the world literature, they identified a median age of onset of 15 years with a range of 9–74 years [2]. This was very similar to the results of the largest single series in which Mackensen et al. reported a median (range) of 15 (6–64) years of age [7]. Indeed TINU may be a relatively common entity in the paediatric population. In a study from Japan, Goda et al. reported that TINU was the second most common diagnosis in children with uveitis [17]. Similarly it can be derived from the Mackensen data that TINU was nearly seven-fold more common as a cause of sudden onset bilateral anterior uveitis in those under the age of 20 than in those above that age; indeed they estimated that 32% of those with the typical uveitis in the younger age group had TINU of which around half were in the ‘definite’ or ‘probable’ categories [7]. It is also worth noting that studies that are based on renal ‘case-finding’ (i.e. that look at the rates of TINU as a subset of all those with biopsy-proven TIN), consistently report higher rates of TINU in children with TIN rather than adults with TIN. For example Li et al., studying a Chinese cohort, found that 31/112 (28%) adults with TIN developed uveitis [18], whereas in their series Perasaari et al. reported that 20/31 (65%) children with TIN developed uveitis [19].

### Female gender may be a risk factor for TINU

There may also be a gender bias in TINU. Although Mandeville reported a 74% (*n* = 98/133) female preponderance they noted that the proportion of male patients being reported was increasing over time. Most series, even recent ones, continue to report a female bias, although the Mackensen study found that 60% of their 33 cases were male. It is also suggested that gender may affect age of onset. Mandeville et al. reported a median age of onset of 14 years in males versus 17 years in females [2], and Mackensen et al. a median age of onset of 15 years in males versus 40 years in females [7].

### Ethnicity does not appear to be a risk factor for TINU

TINU syndrome has not yet been shown to have racial predilection with cases reported across most ethnic groups, and being reported to be present (at 0.2% or higher) in specialist uveitis services across the world including the USA, UK, Germany, Italy, Israel, Japan and Thailand [2]. The possibility of genetic susceptibility is discussed separately below.

## Genetic and environmental factors

### Genetic susceptibility as a risk factor for TINU

Evidence of a genetic predisposition comes from familial clustering and human leucocyte antigens (HLA)-susceptibility studies [19]. Clinical reports include monozygotic twins, siblings and a case of a mother and son diagnosed with TINU several years apart [19–23]; there is also one report of monozygotic twins who both developed interstitial nephritis but with uveitis only occurring in one of them [24].

Several studies have reported on specific HLA associations with TINU syndrome [19, 25–30] but all studies are limited in size and there is significant variation between studies that may reflect the populations sampled. In addition, many earlier reports are based on serological techniques that restricts comparison to later studies [21, 27].

Based on early reports Mandeville et al. suggested that HLA-A2 and -A24 were important antigens associated with this disorder in Japanese subjects, because these 2 antigens had been identified in a majority of Japanese patients with TINU (75%) however both of these specificities are common in the Japanese population [2]. Indeed Matsumoto et al. reported that whereas HLA-A2 and –A24 were present in 32% and 55% of 22 Japanese patients with biopsy proven TINU, this compared to 48% and 64% of 50 healthy Japanese controls [30].

In 2003 Levinson reported on a significant multicenter study of 18 patients with TINU from the USA in which they found that TINU was associated with HLA-DQA1*01, HLA-DQB1*05 and HLA-DRB1*01 with relative risks (RR) of 19.5, 16.3 and 25.5 respectively, and a weaker association with HLA-B14 (RR = 8.5) [25]; caution is needed in interpreting the relative contributions of each of these ‘risk’ alleles due to linkage disequilibrium. The strongest association was with the HLA-DRB1*0102 allele (a subtype of HLA-DRB1*01) which they reported to be present in 13/18 (72%) of TINU patients vs 1.6% of the control population leading to an estimated RR of 167.1; the control rates were based on published rates in North American whites based on 17/18 of the series being of this genetic background. In fact it appears that this allele was only present in 12/18 of that cohort, leading the group to subsequently revise the relative risk of that allele to 46.3 [26].

In a later study Mackensen et al. compared the allelic frequencies noted in this original study, with two clinically relevant cohorts: (1) patients with sudden-onset, anterior bilateral uveitis but without TIN (*n* = 28); and (2) patients with TIN but without uveitis (*n* = 14) [26]. It should be noted that these two comparator groups were conducted in a European population (and the control rates were also based on published European allelic frequencies), but it is striking that the HLA-DRB1*0102 allele was associated with the uveitis cohort (RR = 14.3) but not in those with tubulointerstitial nephritis without uveitis; this uveitis cohort was also associated with HLA-DRB1*08 (RR = 4.0), an allele which had not been found to be associated with the original TINU cohort (*n* = 1/18). Interestingly Reddy et al. reported that 14/15 paediatric patients with unexplained panuveitis had a HLA-DRB1*01-HLADQB1*05 haplotype (identified as being high risk for TINU in the Levinson study) but did not have any evidence of interstitial nephritis, again raising the possibility that some of these alleles are risk factors for uveitis rather than specifically for TINU [29].

In Finland Perasaari et al. conducted a population based study that identified 31 paediatric patients with biopsy-proven TIN, in whom 20 patients were identified as having TINU [19]. This reported a series of novel HLA-associations but did not detect associations with the previously reported ‘TINU susceptibility’ alleles identified by Levinson. The previously identified ‘high risk’ HLA-DRB1*0102 allele is very rare in the Finnish population, and neither it nor any other HLA-DRB1*01 allele was found to be associated with TINU in this cohort. The susceptibility alleles in the Finnish cohort were HLA-DQA1*04:01 (RR = 4.0), HLA-DQA1*01:04 (RR = 6.1), HLA-DRB1*08 (RR = 3.0), and HLA-DRB1*14 (RR = 8.2) [19]. The association between TINU and HLA-DRB1*08 is interesting since, as noted earlier, Mackensen had previously found this to be associated with sudden-onset, anterior bilateral uveitis in the absence of TIN and not associated with TINU itself [26].

Numerous other case reports and small case series have been published in which selected haplotype data is presented [27] but provide little additional contribution to our overall understanding of genetic susceptibility in TINU. All studies vary in their study design, case-finding and population sampled, and there is as yet no consistent evidence to define a susceptibility genotype across populations.

### Drugs as a risk factor for TINU

Two major acquired risk factors have been proposed for TINU: drugs and infections. The main drug groups that have been implicated are non-steroidal anti-inflammatory agents and antibiotics [31–35]. Caution should be exercised when evaluating studies that report associations between these environmental risk factors and the onset of TINU. A number of factors should be borne in mind: (1) most of the studies in this area are retrospective and are subject to recall bias; (2) the proposed risk factors are very common in the general population and yet most studies do not have a control group to provide any comparator for this; (3) the risk factors may co-exist causing difficulties in assessing their relative contributions (e.g. a patient who develops TINU after an infection which has been treated with antibiotics with symptom relief by NSAIDs); (4) the risk factors for TINU may not be identical to the risk factors for TIN without uveitis. The following studies should be considered within this context.

In their 2001 review, Mandeville et al. noted that evaluation of potential risk factors for TINU had been considered for 122 of 133 cases, with positive identification of risk factors in 63 [2]. Antibiotic usage was the commonest reported risk factor (29/122), with NSAIDs the next most common (22/122). In their series of 33 patients with TINU, Mackensen et al. reported that 9/33 had been taking NSAIDs (7/9 were ibuprofen), and 2/33 had been taking antibiotics prior to disease onset; however they concluded that there were no cases of definite drug-induced TINU [7]. In a series of 31 patients from China, Li et al. reported that prior drug usage was identified in 20/31 cases comprising antibiotics (6/31), NSAIDs (1/31), Chinese herbs (1/31) or a combination of drugs (12/31) [18]. In the series of 31 patients from Finland, Perasaari et al. reported 19/31 patients had received antibiotics or non-steroidal anti-inflammatory drugs (NSAIDs) or both, within the two months prior to diagnosis [19].

TIN cohort studies with few or no cases of reported co-existing uveitis, typically comprise older patients and feature a majority (60–70%) postulated to be drug-induced [35, 36], with antibiotics, proton pump inhibitors (PPI) and NSAIDs being the commonest proposed casual agents. Most drug-induced TIN is thought to be a hypersensitivity reaction rather than direct toxicity [35]. Notably, unlike antibiotics and NSAIDs, PPIs have not been linked to TINU, perhaps suggesting distinct pathogenic mechanisms for ocular and renal injury among these different drug classes.

It is interesting that the drugs most commonly reported as a potential precipitant for TIN or TINU are not those for which there is strong evidence of causing isolated drug-induced uveitis. Moorthy et al. reviewed causes of isolated (non-TINU) drug-induced uveitis, using the well-established Naranjo’s criteria to assess likelihood of causality between drugs and adverse reactions [37, 38] Their list of drugs for which there is a ‘definite’ association with inducing uveitis include cidofovir, rifabutin, sulfonamides, bisphosphonates, and both intraocular and topical therapies, but does not include those drugs typically reported in either TIN or TINU. This would suggest that the mechanism of induction of uveitis in the context of TINU may be distinct from other forms of drug-induced uveitis.

### Infection as a risk factor for TINU

In general, infection is thought to be a much less common cause of acute TIN than drug-induced disease. A number of case reports have linked TIN to viral infections including hantavirus, cytomegalovirus, Epstein-Barr virus (EBV), polyoma (BK) virus, adenovirus and HIV. In HIV infection, TIN is normally coexistent with glomerular disease. Tuberculosis is an important cause of TIN that typically exhibits a granulomatous appearance. Mycobacteria cannot usually be identified in renal biopsy by Ziehl-Neelsen staining and empirical treatment may be required after early morning urine cultures have been collected. Granulomatous TIN may also arise from non-infective causes, specifically drug-induced TIN and sarcoidosis. Legionella and histoplasmosis have also been reported to cause TIN but in the main, bacterial and fungal infection is rarely associated with acute TIN.

With regard to TINU, Mandeville et al. noted that infections had been reported in a number of patients, most commonly respiratory tract infections (15 of the 122 for which risk factors had been considered); other sites reported were gastrointestinal, kidney, and other genitourinary sites [2].

Specific infective agents reported as being possibly associated with TINU include tuberculosis [39, 40] systemic toxoplasmosis [41], EBV [42–44] and varicella zoster reactivation [45]. The evidence for the aetiological link here is very variable. In the cases reporting tuberculosis-associated TINU, the *Mycobacterium tuberculosis* does indeed appear to be causative although it is arguable whether these should be classified as TINU given the exclusions regarding underlying systemic disease proposed by Mandeville et al. [2]. In most of the viral-associated TINU the link is much less certain, and may simply be based on the presence of positive serology, such as IgG for EBV, that occurs at a high prevalence in the background population [44]. Most TINU studies do not specifically comment on the presence of preceding infection. Some estimate may be inferred from the frequency of prior antibiotic usage, such as in the studies by Mackensen et al., Li et al. and Perasaari et al. [7, 18, 19].

### Other risk factors for TINU

Various studies have noted the coexistence of other systemic diseases, notably rheumatoid arthritis, hyperthyroidism and parathyroidism; it is possible that they are linked in individual cases due to shared inappropriate immune responses, but it is equally possible that these are coincidental occurrences [2].

The focus of this review on TINU is is on the epidemiological aspects of TINU rather than its pathogenesis, however it should be noted that the increased understanding of one can inform the other: identification of risk factors for TINU through epidemiological studies may help inform our understanding of its pathogenesis; conversely elucidation of the pathogenesis of TINU through basic science studies may improve our understanding of which of the putative risk factors are relevant and how they interact. In this regard the proposal by Tan et al. that modified C-reactive peptide may be a target auto-antigen in *TINU* is of particular interest [46]. Although it needs to be further explored, it can be readily seen how such hypotheses of a final common autoimmune pathway provides an explanation of how different environmental triggers might cause genetically susceptible individuals to develop TINU.

## Conclusion

In this systematic epidemiological review of TINU we have critically appraised current estimates of the incidence and prevalence of this rare disease, and to highlight some of the reasons that different studies can lead to widely differing estimates of these measures. It should be noted that the challenges faced in studying the epidemiology of TINU are common to many other rare diseases. As systematic reporting through national or international registries becomes more common, these estimates for TINU and other rare syndromes should become more precise, although it should also be recognized that there also needs to be consensus around disease definitions and what constitutes the inclusion criteria for registration. This is particularly challenging for syndromes which are primarily based on clinical phenotype (such as most of the uveitic syndromes) rather than those which can be confirmed on the basis of a distinct genotype or have some other sensitive diagnostic test.

We have also used the epidemiological data and the key cohorts identified to consider the risk factors for TINU and how this enables identification of susceptible populations. In such populations (notably in the young with sudden onset bilateral uveitis), it may be relatively common. It is proposed that there is a genetic susceptibility, although it remains unclear the extent to which this is specific to TINU or to uveitis in general, and studies have been somewhat variable in their findings across populations, probably reflecting the relatively small size of even the largest TINU studies. Although a full discussion of the pathogenesis of TINU is beyond the scope of this article, it is relevant to note here that a theory of pathogenesis needs to evaluate and then account for those risk factors that are significantly associated with development of the disease. The current proposal is that TINU arises from an interaction of an environmental trigger (such as a drug or rarely an infection) with a susceptible genetic background, and that this triggers an autoimmune cascade. This will be discussed in our companion review (manuscript in preparation), but it should be recognized that the process is very poorly understood, and requires further investigation. Our epidemiological and descriptive studies that identify ‘association’ must be complemented by immunological studies that can elucidate ‘causation’. In this way we will be able to identify the individuals who are at risk, improve diagnosis within these populations, and translate better understanding of disease to improve our care of these vulnerable patients.

## Abbreviations

AIN: Acute interstitial nephritis
AKI: Acute kidney injury
BK: Bk Polyomavirus
EBV: Epstein Barr virus
GFR: Glomerular filtration rate
HLA: Human leucocyte antigens
NSAID: Non-steroidal anti-inflammatory drugs
PPI: Proton pump inhibitors
SLE: Systemic lupus erythematous
TB: Tuberculosis
TINU: Tubulointerstial nephritis
